# Salicylanilide Diethyl Phosphates as Potential Inhibitors of Some Mycobacterial Enzymes

**DOI:** 10.1155/2014/703053

**Published:** 2014-11-04

**Authors:** Martin Krátký, Eva Novotná, Shalini Saxena, Perumal Yogeeswari, Dharmarajan Sriram, Markéta Švarcová, Jarmila Vinšová

**Affiliations:** ^1^Department of Inorganic and Organic Chemistry, Faculty of Pharmacy, Charles University in Prague, Heyrovského 1203, 500 05 Hradec Králové, Czech Republic; ^2^Department of Biochemical Sciences, Faculty of Pharmacy, Charles University in Prague, Heyrovského 1203, 500 05 Hradec Králové, Czech Republic; ^3^Medicinal Chemistry Research Laboratory, Pharmacy Group, Birla Institute of Technology and Science, Hyderabad 500078, India

## Abstract

Antimycobacterially active salicylanilide diethyl phosphates were evaluated to identify their potential drug target(s) for the inhibition of several mycobacterial enzymes, including isocitrate lyase, L-alanine dehydrogenase (*Mt*AlaDH), lysine *ε*-aminotransferase, chorismate mutase, and pantothenate synthetase. The enzymes are related to the nongrowing state of *Mycobacterium tuberculosis*. Salicylanilide diethyl phosphates represent new candidates with significant inhibitory activity especially against L-alanine dehydrogenase. The most active *Mt*AlaDH inhibitor, 5-chloro-2-[(3-chlorophenyl)carbamoyl]phenyl diethyl phosphate, has an IC_50_ of 4.96 *µ*M and the best docking results. Other mycobacterial enzymes were mostly inhibited by some derivatives but at higher concentrations; isocitrate lyase showed the highest resistance to salicylanilide diethyl phosphates.

## 1. Introduction

The increased number of drug-resistant tuberculosis (TB) cases worldwide and the evidence of recently reported totally drug-resistant strains demonstrate the urgent need for novel therapeutic interventions [1] including innovative antimycobacterial drugs with no cross-resistance to clinically used drugs.

**Figure 1:**
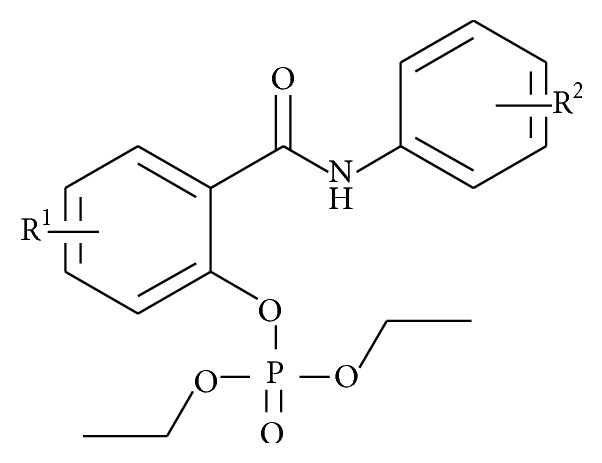
General structure of salicylanilide diethyl phosphates** 1** (diethyl [2-(phenylcarbamoyl)phenyl] phosphates; R^1^ = 4-Cl, 5-Cl, 4-Br; R^2^ = 3-Cl, 4-Cl, 3,4-diCl, 3-Br, 4-Br, 3-F, 4-F, 3-CF_3_, 4-CF_3_).

Recently, salicylanilide diethyl phosphates (diethyl [(2-phenylcarbamoyl)phenyl] phosphates** 1**; Figure 1) have been synthesized as potential antimycobacterial agents with activity in the micromolar range (minimum inhibitory concentrations (MICs) from 1 *µ*M). They inhibit nontuberculous mycobacteria and both drug-susceptible and drug-resistant* Mycobacterium tuberculosis (Mtb)* strains [2]. Previously, some salicylanilide-based derivatives were reported as mild inhibitors of mycobacterial isocitrate lyase (ICL) and methionine aminopeptidase [3]. The exact mechanism(s) of their action as antimicrobial agents has still not been fully elucidated. Therefore, we screened the presented derivatives for new enzymatic targets of* Mtb*, especially those related to the nongrowing state. No inhibitor of the selected enzymes has been established for clinical practice to date.

*Mycobacterium tuberculosis* exhibits a tendency to remain latent or persistent for decades before its activation into symptomatic disease. The bacterium has developed ingenious mechanisms to survive inside a hostile environment and to acquire essential nutrients. These metabolic processes appear to provide potential targets for novel anti-TB agents [4]. Genetic analysis has revealed a set of new potential drug targets in* Mtb*.

Isocitrate lyase (ICL; EC 4.1.3.1) is one of two enzymes comprising the glyoxylate shunt and splits isocitrate into succinate and glyoxylate; this metabolic pathway is absent in vertebrates. ICL is responsible for the persistence of* Mtb* and, additionally, disruption of the* icl* gene attenuated bacterial virulence and adaptation to hypoxia [3]. Based on the fact that salicylanilides and their esters with various acids have been reported as isocitrate lyase inhibitors [3, 5], we evaluated salicylanilide diethyl phosphates** 1** against this enzyme (Table 1).

The mycobacterial L-alanine dehydrogenase (*Mt*AlaDH; EC 1.4.1.1) catalyzes the NADH-dependent reversible oxidative deamination of L-alanine to pyruvate and ammonia [6]. Both L-alanine and D-alanine are important components of peptidoglycan.* Mt*AlaDH plays a key role in the utilisation of carbon and nitrogen sources. It was observed that in the persistent state of the microorganism the gene coding* Mt*AlaDH is upregulated [7, 8].

Lysine *ε*-aminotransferase (*Mt*LAT; EC 2.6.1.36) has been implicated in the mycobacterial stress response and is upregulated by approximately 40-fold in nutrient-starved models designed to mimic the persistent/latent state of TB [9].* Mt*LAT also plays an important role in adaptation to long-term persistence in* Mtb* [10]. In addition, this enzyme has been shown to be upregulated during adaptation to the stationary phase and low-oxygen dormancy [11].

Chorismate mutase (*Mt*CM; EC 5.4.99.5) is another promising selective drug target [12]. This enzyme catalyzes the Claisen rearrangement of chorismate to prephenate in the shikimate pathway, which is the first committed step in the biosynthesis of the aromatic amino acids phenylalanine and tyrosine.

Pantothenate synthetase (*Mt*PS; EC 6.3.2.1) catalyzes the essential adenosine triphosphate-dependent condensation of D-pantoate and *β*-alanine to form pantothenate in bacteria, yeast, and plants. Pantothenate is a key precursor for the biosynthesis of coenzyme A and acyl carrier protein, which are essential cofactors for bacterial growth [13, 14].

We evaluated salicylanilide diethyl phosphates** 1** also against these four mycobacterial persistence-related enzymes as a pilot screening.

## 2. Materials and Methods

### 2.1. Chemistry

The synthesis and characterization of the salicylanilide diethyl phosphates were published previously [2]. Yield of esters synthesized* via* reaction of parent salicylanilides with diethyl chlorophosphate in the presence of triethylamine ranged from 11% up to 78%.

### 2.2. Enzyme Inhibition Measurement

#### 2.2.1. Isocitrate Lyase Assay (ICL1)

Isocitrate lyase activity was assayed according to the protocol reported by Dixon and Kornberg (glyoxylate phenyl hydrazone formation) [15] at 10 *µ*M of the investigated compounds. Isoniazid was employed as a negative control (inhibition of 0%), and 3-nitropropionic acid (3-NP) served as a positive control. A description of the method can be found in the literature [5].

#### 2.2.2. Mycobacterial L-Alanine Dehydrogenase (*Mt*AlaDH) [16] Assay

A reaction mixture consisting of 125 mM glycine/KOH (pH 10.2), 100 mM L-alanine, 1.25 mM NAD^+^, and 6.026 pM of* Mt*AlaDH in a final volume of 200 *μ*L diluted in 125 mM glycine/KOH (pH 10.2) was added to each well of a 96-well plate. The compounds were then added to the plates. The reaction was initiated by the addition of 10 *μ*L enzyme diluted in buffer. Enzymatic activity was measured by the rate of production of NADH that accompanies the conversion of alanine to pyruvate by oxidative deamination [17]. The reaction components, except for* Mt*AlaDH, were mixed in the well and the background reaction was measured;* Mt*AlaDH was then added and the reaction kinetics were monitored. All measurements were performed at 340 nm with a heat-controlled Perkin Elmer Victor V3 spectrophotometer.

#### 2.2.3. Mycobacterial Lysine *ε*-Aminotransferase (*Mt*LAT) [18] Assay

The reaction mixture consisting of 1 mM L-lysine HCl, 1 mM *α*-ketoglutarate, 15 *μ*M pyridoxal-5′-phosphate, and 1.25 pM* Mt*LAT in a final volume of 200 *μ*L diluted in 200 mM phosphate buffer (pH 7.2) was added to each well of a 96-well plate. Compounds were then added to the plates. The reaction was initiated by the addition of 10 *μ*L of* Mt*LAT, diluted in buffer. The mixture was incubated at 37°C for 1 h. The reaction was terminated by the addition of 10% trichloroacetic acid in ethanol. Piperideine 6-carboxylate (P6C) was detected by measuring the colour intensity of its adduct with 2-aminobenzaldehyde spectroscopically at 465 nm. The reaction components except for* Mt*LAT were mixed in the well and the background reaction was measured;* Mt*LAT was then added and the reaction kinetics were monitored. Reactions were carried out at 37°C in a heat-controlled Perkin Elmer Victor V3 spectrophotometer.

One LAT unit (1 U) is the activity that produces 1 *μ*M of P6C per min under these conditions.

#### 2.2.4. Mycobacterial Chorismate Mutase (*Mt*CM) [19] Assay

Reaction volumes of 0.4 mL of chorismate (typically 1 mM) in 50 mM Tris HCl (pH 7.5), 0.5 mM EDTA, 0.1 mg/mL bovine serum albumin, and 10 mM *β*-mercaptoethanol were incubated at 37°C for 5 min. The reaction was started with the addition of 10 *µ*L 5 pM of* Mt*CM (i.e., 185 ng of* Mt*CM equivalent to 12.5 nM final concentration of the dimer based on the molecular mass of 36,948 Da). The reaction was allowed to proceed at 37°C and was terminated after 1 to 5 min with 0.4 mL 1 M HCl. After a further incubation at 37°C for 10 min to convert prephenate, which is formed in the enzymatic reaction, to phenylpyruvate, 0.8 mL 2.5 M NaOH was added. The absorbance of the phenylpyruvate chromophore was read at 320 nm. We set up a blank with no enzyme for every reaction to account for the nonenzymatic conversion of chorismate to prephenate and added the enzyme after the addition of NaOH. The absorbance at 320 nm for the blank varied from 0.1 to 0.3, depending on the concentration of chorismate and the duration of the reaction. A description of the methods can be found in the literature [19].

#### 2.2.5. Mycobacterial Pantothenate Synthetase (*Mt*PS) Screening [20, 21]

Sixty microliters of the PS reagent mix was added, including NADH, pantoic acid, *β*-alanine, ATP, phosphoenolpyruvate, MgCl_2_, myokinase, pyruvate kinase, and lactate dehydrogenase in buffer, to each well of a 96-well plate. The compounds were then added to plates in 1 *μ*L volumes. The reaction was initiated by the addition of 39 *μ*L PS diluted in buffer. The final concentrations in the reaction were 0.4 mM NADH, 5 mM pantoic acid, 10 mM MgCl_2_, 5 mM *β*-alanine, 10 mM ATP, 1 mM potassium phosphoenolpyruvate, and 18 units/mL each of chicken muscle myokinase, rabbit muscle pyruvate kinase, and rabbit muscle lactate dehydrogenase diluted in 100 mM HEPES buffer (pH 7.8), 1% DMSO, and 5 *μ*g/mL PS in a final volume of 100 *μ*L. The test plate was immediately transferred to a microplate reader, and absorbance was measured at 340 nm every 12 sec for 120 sec. Each plate had 16 control wells in the two outside columns, of which 12 contained the complete reaction mixture with a DMSO carrier control (full reaction) and four did not have PS added (background). The per cent inhibition was calculated using the following formula: 100 ∗ 1 − compound rate − background rate/full reaction rate − background rate.

### 2.3. Molecular Docking Studies

The crystal structure of* Mt*AlaDH was obtained from the Protein Data Bank (http://www.pdb.org/, pdb code 2VHW). Water molecules and NAD^+^ were removed, polar hydrogens were added, partial charges were assigned, and the energy of the molecule was minimised using UCSF Chimera software 1.6.2. [22]. The ligand structure was created using CS ChemOffice version 10.0 (CambridgeSoft), and its conformation optimised with the aid of UCSF Chimera 1.6.2 using the Amber force field.

Docking calculations were carried out using Autodock Vina [23]. The three-dimensional affinity grid box was designed to include the full active site of* Mt*AlaDH (box centre: *x* = −58, *y* = 57, and *z* = 8; size of the box 20 points in each direction). The enzyme structure was kept rigid during the docking procedure. The visualisation of enzyme-ligand interactions was prepared using PyMol 1.1r1. [24].

## 3. Results and Discussion

With respect to isocitrate inhibition, most of the evaluated compounds** 1** were inactive at the concentration of 10 *μ*M; some of these molecules displayed the ability to activate the tested enzyme. Only seven derivatives showed very weak inhibition, within the range from 4 to 7%, but without any significant activity. It seems that only halogen monosubstitution of the aniline ring and the presence of 4-bromo or 5-chloro substitution on the salicylic ring retain some inhibitory activity. Salicylanilide diethyl phosphates** 1** failed with respect to finding more efficient ICL inhibitors and when compared with previously described esters [3, 5]. The low inhibition rates observed for these phosphate esters** 1** indicate the importance of the acid used for the esterification of parent salicylanilides in the inhibition results.

To explore other possible target(s) of derivatives** 1**, the inhibitory activity at 50 *µ*M was screened against four other mycobacterial enzymes,* Mt*AlaDH,* Mt*LAT,* Mt*CM, and* Mt*PS (Table 2), which play very important roles in* Mtb* persistence. The investigated compounds showed weak activity against* Mt*PS (the highest activity was approximately 26% for** 1r**) and* Mt*LAT, where only compound** 1s** showed inhibition higher than 50%. More than 50% inhibitory activity against* Mt*CM was found for thirteen compounds, with the highest* in vitro* efficacy for compounds** 1o** and** 1s** (above 60%). The highest percentage activity was found for inhibition of* Mt*AlaDH; compounds** 1s**,** 1q,** and** 1n** had more than 70% inhibition. Therefore, their IC_50_ values were determined and are reported in Table 2. We plotted the graphs of the inhibition rates [%] of 50, 25, 12.5, 6.25, 3.13, and 1.56 *μ*M and calculated IC_50_ values. 5-Chloro-2-[(3-chlorophenyl)carbamoyl]phenyl diethyl phosphate,** 1s**, was found to be the most potent compound against* Mt*AlaDH with an IC_50_ of 4.96 *µ*M. Interestingly, this molecule also exhibited superior inhibition of* Mt*LAT and* Mt*CM, as pointed previously. Its MICs against actively growing* Mtb* H_37_Rv strain were 4–8 *µ*M [2].

Based on the inhibition results, the whole series of salicylanilide diethyl phosphates** 1** was investigated in a molecular docking study to identify possible interactions with amino acid residues in the active site of* Mt*AlaDH. The three-dimensional structure of native* Mt*AlaDH is described as a hexamer formed by three associated dimers of protein subunits. Each subunit consists of two distinct domains, the substrate-binding domain (residues 1–129 and 311–370) and the NAD^+^/NADH binding domain (residues 130–310). These domains are separated from each other by a cleft in which most of the active site amino acid residues are located [16].

**Figure 2:**
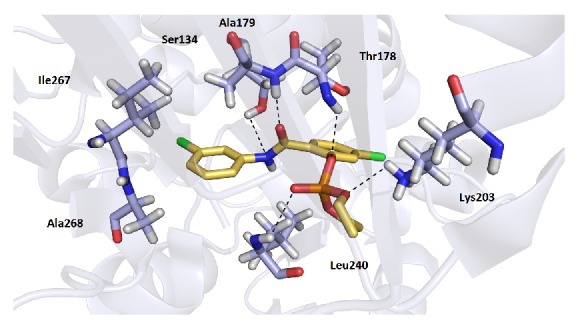
Molecular docking of the** 1s** derivative with H-bonds and hydrophobic interactions with amino acid residues of* Mt*AlaDH.

**Figure 3:**
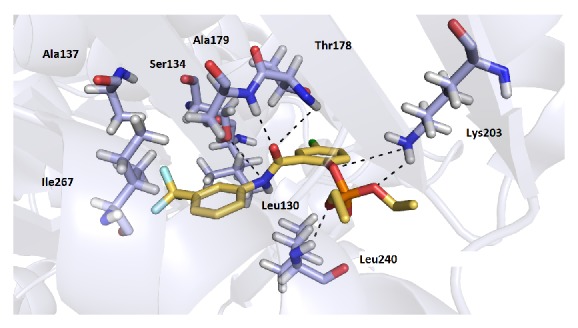
Molecular docking of the** 1q** derivative with trifluoromethyl group pointing into hydrophobic cavity comprising residues Leu130, Ala137, and Ile267.

The top-scoring orientations of the compounds were located in the cavity normally occupied by NAD^+^ (Figure 2). All of the compounds in the series displayed a similar conformation in the active site. Since structural differences of the compounds** 1** are very small, the docking studies enabled us only to predict possible orientation in the active site of the enzyme, but the influence of different position of substitution on the activity is not clear. In general, the orientation of all of the compounds suggests possible H-bond interactions, predominantly with two amino acid residues, Thr178 and Ala179. In addition, some of the compounds showed another H-bond interaction with Ala238, Leu240, Ser134, and Lys203. The most active compound in the series, 5-chloro-2-[(3-chlorophenyl)carbamoyl]phenyl diethyl phosphate** 1s** (IC_50_ 4.96 *µ*M), exhibited H-bond interactions with residues Ser134, Thr178, Ala179, Lys203, and Leu240 and additional hydrophobic interactions with amino acid residues Ile267 and Ala268 (Figure 2), which resulted in one of the highest affinities for the enzyme (docking score −7.8 kJ/mol). Good docking results were also observed for compounds with a trifluoromethyl substitution in the aniline part (**1e**,** 1f**,** 1q**,** 1r**,** 1z,** and** 1zz**). These compounds demonstrated the same H-bond interactions as** 1s**. Additionally, the trifluoromethyl group is positioned in a small cavity occupied by hydrophobic amino acid residues (Leu130, Ala137, and Ile267), and the potential to form hydrophobic interactions may cause a higher binding affinity of these derivatives for the enzyme (Figure 3). However, another mechanism of the salicylanilide action may participate together with inhibition of* Mt*AlaDH.

We have found that there is not a direct relationship of* in vitro *MICs of salicylanilide diethyl phosphates against actively growing* Mtb* and the inhibition of five presented enzymes, especially when the suppression of these enzymes should affect especially persistent mycobacterial subpopulation. The results confirm the fact that salicylanilide derivatives share a complex mechanism of action with more molecular/cellular targets.

## 4. Conclusions

To identify potential TB drug target(s) of salicylanilide diethyl phosphates, they were evaluated against five mycobacterial enzymes related to dormancy. Most of the compounds exhibited significant inhibition, especially against* Mt*AlaDH with IC_50_ of 4.96 *μ*M or higher. Additionally,* Mt*LAT and* Mt*CM were affected considerably by diethyl phosphates. Salicylanilide derivatives have multiple mechanism of action and the activity against these enzymes only contributes to their antimycobacterial activity. There is no clear correlation between MICs against actively growing* M. tuberculosis* and inhibition of enzymes, which are important predominantly or exclusively for persistent state.

Our data represent the results of enzyme inhibition screening. Further studies to verify that these compounds are true inhibitors of* Mt*AlaDH are required (e.g., inducing drug-resistant mutants and identification of possible mutations, cocrystallisation of the enzyme with an inhibitor, etc.). Based on structural similarity, related and analogous derivatives may be designed and evaluated as prospective inhibitors of this enzyme.

## Figures and Tables

**Table 1:** ICL inhibition activity of selected salicylanilide diethyl phosphates **1**.

	R^1^	R^2^	% ICL inhibition at 10 *μ*M (±standard deviation)
**1a**	4-Br	3-F	7 ± 2.45
**1h**	4-Br	4-Br	6 ± 2.41
**1s**	5-Cl	3-Cl	6 ± 0.83
**1u**	5-Cl	3-F	4 ± 0.50
**1v**	5-Cl	4-F	6 ± 0.92
**1w**	5-Cl	4-Br	6 ± 1.00
**1x**	5-Cl	4-Cl	6 ± 1.31

**3-Nitropropionic acid**			25 ± 4.1
**Isoniazid**			0

Esters, which are not reported here, had no ICL inhibition.

**Table 2:** Mycobacterial enzyme activity inhibition results.

Comp. code	R^1^	R^2^	% inhibition at 50 *μ*M against L-*Mt*AlaDH	L-*Mt*AlaDH IC_50_ (*μ*M)	% inhibition at 50 *μ*M against *Mt*LAT	% inhibition at 50 *μ*M against *Mt*CM	% inhibition at 50 *μ*M against *Mt*PS
**1a**	4-Br	3-F	69.94	39.75	7.80	40.50	7.47
**1b**	4-Br	3-Cl	59.46	41.12	18.73	43.50	3.89
**1c**	4-Br	4-Cl	39.35	>50	29.10	48.31	13.25
**1d**	4-Br	3,4-di-Cl	59.02	31.92	30.27	41.30	20.54
**1e**	4-Br	3-CF_3_	42.08	>50	40.20	53.20	17.76
**1f**	4-Br	4-CF_3_	69.28	34.73	26.15	52.09	11.33
**1g**	4-Br	3-Br	22.62	>50	12.42	46.07	18.76
**1h**	4-Br	4-Br	34.35	>50	18.25	38.19	18.33
**1i**	4-Br	4-F	54.58	15.58	2.90	46.67	5.41
**1j**	4-Cl	3-Cl	12.30	>50	29.30	54.56	3.64
**1k**	4-Cl	4-Cl	18.73	>50	38.00	43.78	14.74
**1l**	4-Cl	3-F	61.87	23.11	8.06	50.75	17.93
**1m**	4-Cl	4-F	57.70	42.26	20.12	49.33	7.76
**1n**	4-Cl	4-Br	**70.64**	29.17	17.29	50.19	6.57
**1o**	4-Cl	3,4-di-Cl	44.46	>50	18.24	**60.96**	23.01
**1p**	4-Cl	3-Br	54.64	36.11	19.13	37.41	20.59
**1q**	4-Cl	3-CF_3_	**73.88**	36.32	19.05	50.02	11.53
**1r**	4-Cl	4-CF_3_	47.28	>50	23.18	50.05	**26.25**
**1s**	5-Cl	3-Cl	**73.94**	**4.96**	**53.64**	**60.12**	11.18
**1t**	5-Cl	3-Br	41.51	>50	12.86	57.58	5.04
**1u**	5-Cl	3-F	59.85	39.20	23.42	52.36	22.22
**1v**	5-Cl	4-F	69.07	34.47	37.29	52.05	17.28
**1w**	5-Cl	4-Br	14.97	>50	40.28	52.84	14.51
**1x**	5-Cl	4-Cl	58.62	46.51	20.18	53.79	11.17
**1y**	5-Cl	3,4-di-Cl	26.30	>50	28.17	56.65	12.38
**1z**	5-Cl	3-CF_3_	58.32	17.82	10.01	43.49	4.51
**1zz**	5-Cl	4-CF_3_	64.82	37.49	19.83	54.09	8.26

The best results for each enzyme are shown in bold.
